# Biochemical Studies of Sulphocyanates

**Published:** 1913-02-15

**Authors:** 


### BIBLIOGRAPHY.

BIOCHEMICAL STUDIES OF SULPHOCYANATES BY MAX KAHN, NEW YORK CITY.—Another contribution to the study of the saliva has been made by Dr. Max Kahn of New York City in a pamphlet entitled "Biochemical Studies of Sulphocyanates." It is the report of work done by him in the University of Columbia, under Dr. Wm. Gies, and was presented as a thesis for the degree of Doctor of Philosophy. It is published in pamphlet form by the Eschenbach Printing Co., of Easton, Pa. The subject matter covers 83 pages.

The author has devoted the first 32 pages to a careful and painstaking review of the history of work and publications on sulphocyanates in the body. He states the various theories, and makes a very fair statement of the present knowledge of the sulphocyanates in the saliva.

The second chapter deals with the criticism which Bunting made as to the validity of the ferric chloride test in the saliva. The author has tested out the various substances which might interfere with the reaction both by the ordinary test and the ether test as suggested by Bunting. His conclusions are that "the ferric chloride colorimetric test for the thiocyanates in saliva is inexact and unreliable." Also that "various medicinal substances and various chemical compounds that are the result of decomposition of proteids and carbohydrates may, if excreted in the saliva, give a very marked coloration when treated with ferric chloride, and thus convey the impression that the amount of thiocyanate is very large." He gives in the next chapter descriptions of the Munk Gravimetric Method and the Rupp, Schied, and Thiel Iodometric Method for determining sulphocyanates.

In the succeeding chapters the author has made a study of the occurrence of thiocyanates in the various tissues and excretions of the animal body. He states that the liver contains more of the salt than any other gland in the body. That it is secreted by the liver into the bile and is poured into the small and large intestines. He also found it in the blood, but not in the pancreas, thymus, thyroid, or testicles. The dog had no thiocyanates in the salivary gland or saliva. He further found that the amount of these cyanates in the body was directly dependent upon the amount of amino-acids and nitrates ingested, and concludes that they were the result of proteid metabolism and essentially an excretion.

In his concluding chapter he reports work relative to the effects of the administration of KSCN to plants and animals. The animals used were frogs and dogs. He states that "Potassium sulfocyanate is toxic to both plants and animals. Its toxicity is so marked that indiscriminate dispensation of the substance to patients is dangerous. The growth of moulds is enhanced by potassium sulfocyanate. Small biological quantities of KSCN have no inhibiting effect on bacteria. Yeast fermentation is uninhibited or stimulated by potassium sulfocyanate. The souring of milk is inhibited by large (relatively) amounts of thiocyanate.

The work as reported by the author seems to have been carried out in a very scientific and thorough manner. It throws a good deal of light upon the nature, sources, and occurrence of thiocyanates in the saliva and is a very valuable contribution to the subject.

